# Intermittent claudication and severe renal artery stenosis are independently associated in hypertensive patients referred for renal arteriography

**DOI:** 10.6061/clinics/2017(07)04

**Published:** 2017-07

**Authors:** Thiago Andrade Macedo, Luciano Ferreira Drager, Rodrigo Pinto Pedrosa, Henrique Cotchi Simbo Muela, Valeria Costa-Hong, Luiz Junia Kajita, Luiz Aparecido Bortolotto

**Affiliations:** I Unidade de Hipertensao, Divisao de Cardiologia, Instituto do Coracao (InCor), Hospital das Clinicas HCFMUSP, Faculdade de Medicina, Universidade de Sao Paulo, Sao Paulo, SP, BR; II Laboratorio de Hemodinamica, Divisao de Cardiologia, Instituto do Coracao (InCor), Hospital das Clinicas HCFMUSP, Faculdade de Medicina, Universidade de Sao Paulo, Sao Paulo, SP, BR

**Keywords:** Intermittent Claudication, Renal Artery Stenosis, Renal Angiography

## Abstract

**OBJECTIVE:**

The purpose of this study was to evaluate the association between the presence of clinical symptoms of peripheral artery disease and severe renal artery stenosis in patients referred for renal angiography.

**METHOD:**

We included 82 patients with clinical suspicion of renovascular hypertension and performed an imaging investigation (renal Doppler ultrasound and/or renal scintigraphy) for possible renal artery stenosis. All patients underwent renal arteriography and were examined for peripheral artery disease based on the presence of intermittent claudication and ankle-brachial index test results. Severe renal artery stenosis was defined as a lesion causing 70% obstruction.

**RESULTS:**

Severe renal artery stenosis was present in 32 of 82 (39%) patients. Patients with severe renal artery stenosis were older (63±12 *vs* 56±12 years, *p*=0.006), had more intermittent claudication (55 *vs* 45%, *p*=0.027), and had a greater prevalence of an ankle-brachial index <0.9 (44% *vs* 20%, *p*=0.021) than patients without severe renal artery stenosis. Multivariate logistic regression analysis showed that the presence of intermittent claudication was independently associated with renal artery stenosis ≥70% (OR: 3.33; 95% CI 1.03–10.82, *p*=0.04), unlike the ankle-brachial index, which showed no association (OR: 1.44; 95% CI 0.37–5.66, *p*=0.60).

**CONCLUSION:**

Intermittent claudication is independently associated with severe renal artery stenosis (≥70%) in patients clinically suspected of having renovascular hypertension.

## INTRODUCTION

Atherosclerotic renal stenosis is a significant cause of secondary arterial hypertension and is strongly associated with cardiovascular events 1,2, independent of the degree of hypertension 3,4. Previous studies have shown the coexistence of renal artery stenosis (RAS) with atherosclerosis at other vascular sites 5-7 and high blood pressure is linked to an increased incidence of peripheral artery disease (PAD) 8. Most patients with PAD have no symptoms but do experience intermittent claudication (IC), the main symptom of PAD, which is frequently underdiagnosed 2 in patients with hypertension. During the clinical investigation, the presence of symptoms suggestive of PAD should be further investigated using the ankle-brachial index (ABI) test. This test, besides being an important diagnostic tool, provides significant information about the presence of subclinical atherosclerosis 2 and the increased risk of cardiovascular morbidity and mortality 3. Furthermore, in the presence of vascular risk factors such as hypertension, the detection of asymptomatic PAD may represent the need for a change from primary to secondary prevention, resulting in more rigorous treatment strategies.

Evidence has shown that RAS is a predictor of coronary artery disease (CAD) and a marker of diffuse atherosclerosis 9-11. In a previous study, the authors identified predictors of serious CAD (≥70%) in patients with clinical suspicion of severe RAS (≥70%). Severe RAS is a strong predictor of serious CAD independent of angina, and dual investigation should be considered in patients referred for renal angiography 9. The purpose of our study was to determine whether IC could be a clinical predictor of severe RAS in hypertensive patients referred for renal angiography because the relationship between severe RAS and the presence of clinical symptoms of PAD is not clear.

## MATERIALS AND METHODS

All subjects included in the study had established hypertension and suspicion of RAS based on clinical data. One or more of the following conditions were considered: resistant hypertension (sustained hypertension despite the use of 3 different classes of antihypertensive agents), previous hypertensive pulmonary edema, congestive heart failure, malignant hypertension or progressive renal failure. Subjects were referred to the Hypertension Unit of the Heart Institute (InCor) - Hospital das Clínicas da Faculdade de Medicina da Universidade de São Paulo over a 2-year period. The procedures were conducted in accordance with institutional guidelines, and the institutional review committee approved the protocol (1125/07 – Ethics Committee for Analysis of Research Projects HC-FMUSP). All patients gave written informed consent.

Patients were selected whose angiography would be useful for the diagnosis of significant RAS and according to the risk of RAS based on clinical data and additional diagnostic noninvasive imaging that suggested the presence of RAS as follows: decreased renal perfusion by renal scintigraphy, increased renal blood flow velocity (≥180 cm/s) observed on Doppler ultrasound of the renal arteries, and/or magnetic resonance angiography of renal arteries suggesting stenosis. Patients with known PAD (prior ABI <0.9, peripheral lower extremity vascular procedures, or major lower extremity amputation) or RAS detected by prior renal angiography were excluded.

Risk factors were measured at each clinical examination and included age, resting blood pressure, smoking status, presence of diabetes, and measurement of serum cholesterol levels. Blood tests were performed to measure total cholesterol, low-density lipoprotein cholesterol, high-density lipoprotein cholesterol, and serum creatinine. The diagnosis of hypercholesterolemia was based on whether the patients had been prescribed cholesterol-lowering agents or had a fasting total cholesterol level >200 mg/dL. Patients were considered to have diabetes mellitus if dietary or pharmacological interventions were required to maintain normal blood glucose levels (<126 mg/dL) or if they were using specific medications. Blood pressure (BP) was measured using an automatic digital sphygmomanometer (OMRON-705CP, Japan) after the patient had been in a resting position for 5 minutes. The classification of BP was based on the average of 2 readings taken by the examining physician and according to the recommendations of the Eighth Joint National Committee on High Blood Pressure 4.

Evaluation for the presence of IC was based on a structured history and questions from the Edinburgh Claudication Questionnaire 5. Patients with a positive questionnaire (6 answers) were diagnosed as having IC. The clinical evaluation also included the ABI measured with the patient in the supine position after a 10-minute rest. A portable vascular Doppler device (DV 610, Medmega, SP, Brazil) and a mercury sphygmomanometer with an appropriate cuff size for the patient’s arm circumference were used for measurements. Systolic blood pressure measurements were performed in the following order: right brachial artery, right dorsalis pedis artery, right posterior tibial artery, left dorsalis pedis artery, left posterior tibial artery, and right brachial artery. The ABI was calculated for each lower limb to determine the ratio between the highest ankle pressure (dorsalis pedis or posterior tibial) and highest blood pressure (right or left arm) 6. The lowest ABI value obtained was used for the analysis of the results (right or left ABI).

### Evaluation of renal arteries

All patients underwent renal angiography after a detailed medical history and complete clinical examination were completed. For a statistical comparison of clinical and laboratory variables, the study population was divided into 2 groups, according to the presence (≥70%) or absence (<70%) of severe renal obstruction verified by renal angiography. Serum creatinine levels were determined twice during the evaluation, 1 day before the procedure and 3 days afterwards. The glomerular filtration rate (GFR) was estimated with the Modification of Diet in Renal Disease (MDRD) formula 7. To prevent nephropathy induced by iodinated contrast, patients who had an estimated GFR (eGFR) below 60 mL/min/m^2^ received a normal saline infusion (1 mL/kg/h) before and 24 h after the procedure plus 600 mg of oral *N*-acetyl-cysteine twice daily and low-osmolar contrast for the procedure, as previously described 8.

### Statistical analysis

Data were analyzed using SPSS 20.0 statistical software. Descriptive analysis was used to define the study population. Parametric data are expressed as the mean ± standard deviation, and nonparametric data are expressed as the median followed by the interquartile range or as percentages, when appropriate. Student’s *t* test for independent samples and the Mann-Whitney U test were used to compare quantitative variables of groups with and without significant RAS. A Chi-squared test was used to analyze qualitative variables, and the Fisher correction was used when necessary. Univariate and multiple logistic regression analyses were used to determine the factors associated with significant RAS in the entire population. Variables with a value of *p*<0.1 in the univariate analysis were included in multivariate models. A *p* value <0.05 was considered significant.

## RESULTS

We evaluated 82 patients with clinical suspicion of RAS, consisting of 62% females, with a mean age of 59±13 years. The main characteristics of the total sample and groups categorized by the presence or absence of severe RAS are shown in Table 1. A high frequency of diabetes, IC, and smoking were observed. Severe RAS diagnosed by renal arteriography was present in 32 of 82 (39%) patients. The prevalence of atherosclerosis risk factors was compared between groups (RAS <70% and RAS ≥70%). Patients with severe RAS were older (63±12 *vs* 56±12, *p*=0.006) and had a greater prevalence of IC (55% *vs* 45%; *p*=0.027) and ABI <0.9 (44% *vs* 20%, *p*=0.021). No significant differences were observed between the 2 groups concerning diabetes, smoking status, angina, blood pressure, renal function, and dyslipidemia. The average contrast volume used in renal arteriography was 110 mL. Only 7 (8.5%) patients had a transient and mild/moderate increase in creatinine levels suggestive of contrast-induced nephropathy. Dialysis was not required, and no patient developed serious complications during renal arteriography. In the univariate analysis, the variables that were significantly correlated with the presence of severe RAS included age, ABI, and IC (Table 2). In the multivariate logistic regression analysis, the presence of IC was an independent variable associated with severe RAS (OR: 3.33, 95% CI: 1.03–10.82, *p*=0.04).

## DISCUSSION

The present study produced the following important findings: a) in patients with hypertension who were referred for renal angiography under clinical suspicion of renovascular hypertension, the frequencies of IC and an ABI <0.9 were 35% and 28%, respectively; and b) a validated clinical parameter (presence of IC defined by a standardized questionnaire) was independently associated with severe RAS in patients with hypertension. Atherosclerotic RAS is a clinical condition frequently observed in patients with multiple cardiovascular risk factors and concomitant atherosclerosis in other arterial beds 10,11. Because atherosclerosis is a systemic disease, it is conceivable that a significant proportion of patients with hypertension who have confirmed RAS also have PAD; however, current evidence is scant, even though PAD has a high prevalence 12 and is associated with increased cardiovascular morbidity and mortality 13. Although IC is the symptomatic expression of PAD, approximately 50% of patients with IC remain asymptomatic 14. Conversely, we found a high prevalence (35%) of IC among patients with suspicion of RAS, and IC was more prevalent in the group with RAS ≥70% than in the group without severe RAS (55% *vs* 45%, *p*=0.027). IC has a significant effect on quality of life and is strongly associated with impaired functional capacity 15. In addition, IC is related to a twofold to fourfold increased risk of mortality, predominantly from cardiovascular disease 16, and its presence is a predictor of higher rates of myocardial infarction, stroke, and disability in older adults 17. Similar to the elevated prevalence of the clinical presentation of IC, we found an elevated prevalence of an ABI <0.9 (29%). This parameter was observed more frequently in patients with severe RAS (44% *vs* 20%, *p*=0.021) and is equally associated with an unfavorable prognosis as described in previous studies 6,13,18.

The clinical expression of severity in the group with RAS ≥70% can be verified by uncontrolled blood pressure (SBP median=149 mmHg) despite patients’ use of 4 different classes of antihypertensive medications (Table 1). The prevalence of diabetes mellitus in our study was 44%, which is higher than that reported in other studies of patients with PAD (between 20 and 29%) 19. The group with severe RAS was older and possibly had more diffuse and serious atherosclerosis, which could explain the higher prevalence of PAD 20. We did not find studies that investigated the prediction of RAS based on the presence of PAD defined by clinical parameters or the ABI.

The main strength of the present investigation was the systematic evaluation of IC symptoms and PAD in consecutive patients diagnosed according to the gold standard method used for identifying RAS. We found a potential role for IC symptoms, which can be easily identified when the clinical history is obtained, for predicting severe RAS in high-risk patients. This finding may reinforce the indication of RAS investigation as important in patients with resistant hypertension. Some limitations should also be addressed. First, the sample size was small, which is primarily explained by our single-center study design, and we only investigated patients at high risk, suggesting the presence of RAS. Second, the selection of patients with hypertension at high risk for atherosclerotic RAS could overestimate the number of individuals affected by atherosclerosis in other vessels, including the peripheral arteries. However, both limitations are related to the standard indications for investigating RAS *per se* and could partially explain the high rate (35%) of IC in selected patients despite of prevalence of severe RAS in the total group.

The presence of IC was independently associated with severe RAS in patients suspected of having renovascular hypertension based on clinical evidence.

## AUTHOR CONTRIBUTIONS

Macedo TA conceived and designed the work, wrote the draft and contributed to the correction of the manuscript, was responsible for the acquisition, analysis and interpretation of data for the work, managed the literature searches, conducted research procedures, supervised the work, agreed to be accountable for all aspects of the work and to answer questions related to the accuracy or integrity of any part of the work and approved the final version of the manuscript to be published. Drager LF conceived and designed the work, wrote the manuscript draft and contributed to the correction of the manuscript, supervised the work and approved the final version of the manuscript to be published. Muela HC, Pedrosa RP, Costa-Hong V and Kajita LJ were responsible for the acquisition, analysis and interpretation of data for the work, managed the literature searches, conducted research procedures and approved the final version of the manuscript to be published. Bortolotto LA conceived and designed the work, wrote the draft and contributed to the correction of the manuscript, was responsible for the acquisition, analysis and interpretation of data for the work, managed the literature searches, supervised the work, agreed to be accountable for all aspects of the work and to answer questions related to the accuracy or integrity of any part of the work, approved the final version of the manuscript to be published. All authors read and approved the final manuscript and have participated sufficiently in the work to take public responsibility for appropriate portions of the content.

## Figures and Tables

**Table 1:** Clinical characteristics of the 82 patients according to the presence of severe renal artery stenosis.

Variables	Total (n=82)	RAS (<70%) (n=50)	RAS (≥70%) (n=32)	*p* value
**Age, years**	59±13	56±12	63±12	0.006*
**Female, %**	62	62	66	0.96
**Mulatto, %**	40	34	50	0.32
**Diabetes mellitus, %**	44	42	47	0.66
**Smoking, %**	32	30	34	0.68
**Angina, %**	26	28	22	0.55
**IC, %**	35	45	55	0.027*
**SBP, mmHg**	149±29	146±31	153±25	0.29
**DBP, mmHg**	78±12	77±13	80±11	0.18
**Antihypertensives, n**	4 (3-5)	5 (3.7-5)	4 (3-5)	0.18
**Serum creatinine, mg/dL**	1.4 (1.1-2.3)	1.38 (1.0-2.3)	1.51 (1.1-2.2)	0.49</emph>
**eGFR <60 mL/min/m2, %**	63	58	72	0.20
**ABI <0.9, %**	29	20	44	0.021*
**LDL, mg/dL**	110±35	107±32	113±40	0.46
**HDL, mg/dL**	40 (35-48)	41 (36-48)	36 (32-46)	0.09
**Triglycerides, mg/dL**	134 (95-178)	132 (98-171)	153 (91-202)	0.31

RAS: renal artery stenosis; IC: intermittent claudication; SBP: systolic blood pressure; DBP: diastolic blood pressure; eGFR: estimated glomerular filtration rate; ABI: Ankle-brachial index; LDL: low-density lipoprotein cholesterol; HDL: high-density lipoprotein cholesterol.

**Table 2:** Unadjusted and adjusted odds ratio variables in patients according to severe renal artery stenosis (≥70%).

Univariate			Multivariate	
Variables	OR (CI)	*p* value	OR (CI)	*p* value
**Age**	1.05 (1.01-1.10)	0.009	1.26 (0.97-1.08)	0.70
**ABI**	3.11 (1.16-1.01)	0.009	1.44 (0.37-5.66)	0.60
**IC**	2.85 (1.11-7.27)	0.029	3.33 (1.03-10.82)	0.04*

ABI: Ankle-brachial index; OR: odds ratio; CI: confidence interval; IC: intermittent claudication.

* Adjusted for age, ABI, and IC.
